# Dr. Dyce, on an Old Popular Remedy for the Tooth-Ach

**Published:** 1799-10

**Authors:** William Dyce

**Affiliations:** Aberdeen


### *To the Editors of the Medical and Physical Journal.*

Gentlemen,

The remedies employed by the lower class of people for the cure of diseases, if carefully examined, and their effects properly explained, would afford considerable amusement to medical men, and perhaps be attended with some kind of instruction; but there would be some difficulty in procuring satisfactory information relative to every remedy which they use; some of them being handed down from generation to generation, as carefully as if it were a landed property, and the knowledge of the preparation and use is known only to the family to whom it belongs. In such a case, therefore, fore, little advantage could be expected; except, from stating the effects which are produced from such means, we are able to imitate these with other remedies perhaps more efficacious.

The remedies which are used by the common people of this city for the cure of the tooth-ach, are very various; one of them, however, has appeared to many so extraordinary, having been practised only lately, that I must beg leave to mention it, as well as the explanation given by those who apply it.—I should first mention, that their idea of the cause of this disease is from a worm, or worms, being engendered in the tooth affected, and from this the pain is actually called the *worm*, which, unless this worm be dislodged, cannot be cured. The remedy I allude to is well calculated to support this idea, and very apt to deceive a superficial observer. It is applied in this way:—a small quantity of the seed of some plant is put into a metalic tube, shaped like a tobacco-pipe, in the bowl of which is put the seed, fire is then applied to it; the smoke which issues from the seeds is directed, by means of the tube to the hollow tooth, and being very acrid, causes a prodigious increase of secretion of saliva, which being preserved in a bason, and afterwards viewed with a magnifier, or even with the naked eye, is found to contain a quantity of small white threads, not unlike the ascarides, and these are said to be the worms from out of the tooth: a proof more convincing than ocular demonstration could scarcely be adduced, and of course, little reasoning was necessary to confirm the hypothesis; for facts being stubborn things, it seemed necessary to examine these *worms*, but nothing satisfactory appearing from them, it was most readily *swallowed* by those who had paid little attention to the construction of the human frame.

The only way that seemed to me probable, to explain this uncommon appearance was, that this particular species of seed (which, by the bye, they keep a secret), by having heat applied to it, thereby expelling a small quantity of water from those seeds that are nearest the heat, and softening those above, readily separates its fibrous germs, which, being carried through the tube by the condensed moisture, fall into the mouth, and are there carried off by the saliva into the bason, and are then shewn as the cause of all their distress.

The plant from which these seeds are produced, I have never been able to ascertain; but on looking into some old books, I found the following particulars:—"This peyne doth come eyther by an humour discending out of the head to the teeth or gummes, or it may come by corroding or eating of wormes, or it may come by drinking of hote wynes, eating of hote spices,

ſpices, or eating of hote apples, peares, and ſuch lykes, or it may come of a hote liver or ſtomake.

A REMEDY.

"First purge the head with pilles of cochæ, and uſe gargaries, and if it do come of any cold cauſe, chew in the mouth divers times, the rote of horehound. And if it come by wormes, make a candell of waxe with henbane ſeeds, and light it, and let the perfume of the candell enter into the tooth, and gape over a diſh of cold water, and then may you take the wormes out of the water, and kill them on your naile; the worme is little greater than the worme in a man's hand. And beware of pulling out any tooth, for pul out one, and pul out more. To mundify the teeth, waſhe them every morning with cold water, and a little roche alome*."

---

From this extract, therefore, we find that the idea of worms being the cauſe of tooth-ach is of pretty old date; the book from which it is copied, was printed at London in the year 1575, and is intituled "*The Breviarie of Health; wherein doth follow, remedies for all maner of ſickneſſes and diſeaſes, the which may be in man or woman. Expreſſing the obſcure termes of Greke, Araby, Latin, Barbary, and Engliſh, concerning Phyſicke and Chirurgerie. Compiled by* ANDREWE BOORDE, Doctor of Phiſicke, an Engliſhman."

This book, which is a ſmall quarto, and printed in the black letter, ſeems to have been entirely neglected, but as I find ſome account of the author written in the laſt page of the book, I have copied it, and is as follows:—

"Andrewe Boorde practiſed phyſic in Hampſhire, and was a man of great ſuperſtition, and of a weak and whimſical head. He frequented fairs and markets, and harangued the populace in public; and, to uſe the words of one of his contemporaries, "he made humourous ſpeeches, couched in ſuch "language as cauſed mirth, and wonderfully propagated his fame. From "the Doctor's method of uſing ſuch ſpeeches at markets and fairs, it came "that in after times, thoſe who imitated the ſame humourous, jocoſe lan-"guage, were ſtiled *Merry Andrews*."

Dr. Boorde was author of "The merry tales of the Wiſe Men of Gotham"—"The Introduction of Knowledge, a Poem"—"The Miller of Abington," a poor imitation of Chaucer's Miller's Tale.—"The Principles of Aſtronomical Prognoſtications"—"The Doctrine of Health"—"The Promptuary of Medicine"—The Doctrine of Urins." He lived in the days of

* This method of curing the tooth-ach has long been practiſed in many parts of England. Some uſe cummin-seeds, ſome henbane, others aromatic herbs with a little salt, &c.

of HENRY VIII, EDWARD VI, and Queen MARY. Having been once a Carthusian, he continued ever after to profess celibacy, to drink water, and to wear a shirt of hair. The title-page of his "Introduction of Knowledge" runs thus: "The first boke of the Introduction of Knowledge, the which doth teach a man to speake parte of al maner of languages, and to know the usage and fashion of al maner of countryes, and for to know the most parte of al maner of coynes of money, the which is currant in every region." From this flaming title it appears, that the art of puffing was early known to authors and booksellers.

By the first opportunity, I intend to send you a paper on the instruments at present used in the practice of midwifery, with a description of a new one. I have lately tried the external application of opium in nephritic complaints, as well as ol. tereb. and æther, with the same good effects as Dr. WILLICH mentions *.

I am, Gentlemen,

ABERDEEN, Your's sincerely,

* See our *Journal*, No. V. page 507.

